# AQUA Mutant Protein Quantification of Endomyocardial Biopsy-Sized Samples From a Patient With Hypertrophic Cardiomyopathy

**DOI:** 10.3389/fcvm.2022.816330

**Published:** 2022-02-21

**Authors:** Edgar Becker, Antonio Francino, Andreas Pich, Andreas Perrot, Theresia Kraft, Ante Radocaj

**Affiliations:** ^1^Institute for Molecular and Cell Physiology, Hannover Medical School, Hannover, Germany; ^2^Cardiology Department, Hospital Clinic/IDIBAPS, University of Barcelona, Barcelona, Spain; ^3^Institute of Toxicology, Hannover Medical School, Hannover, Germany; ^4^Experimental and Clinical Research Center, Charité-Universitätsmedizin Berlin, Berlin, Germany

**Keywords:** protein quantification, AQUA, mass spectrometry, heterozygous mutation, mutant protein fraction, hypertrophic cardiomyopathy, endomyocardial biopsy

## Abstract

In genetic diseases like hypertrophic cardiomyopathy, reliable quantification of the expression level of mutant protein can play an important role in disease research, diagnosis, treatment and prognosis. For heterozygous β-myosin heavy chain (β-MyHC) mutations it has been shown that disease severity is related to the fraction of mutant protein in the myocardium. Yet, heart tissue from patients with genetically characterized diseases is scarce. Here we asked, if even in the case of small endomyocardial biopsies, single quantifications produce reliable results. Myocardial samples were taken from four different regions of an explanted heart of a patient with hypertrophic cardiomyopathy carrying point mutation p.Gly716Arg in β-MyHC. From both, large samples (15 mg) and small, endomyocardial biopsy-sized samples (≤ 1 mg) myosin was extracted and enzymatically digested to yield a specific peptide of interest that allowed to distinguish mutant and wild-type β-MyHC. Absolute quantification by mass spectrometry (AQUA) of the peptide of interest was performed repeatedly for both sample sizes to determine the fraction of mutant β-MyHC. Fractions of mutant β-MyHC (32% on average) showed only small differences between the four cardiac regions and for large and small samples. The standard deviations were smaller than five percentage points for all cardiac regions. The two quantification methods (large and small sample size) produce results with comparable accuracy and precision. Consequently, with our method even small endomyocardial biopsies allow reliable protein quantification for potential diagnostic purposes.

## Introduction

Hypertrophic cardiomyopathy (HCM) is often familial and in most mutation-positive cases caused by heterozygous mutations in sarcomeric proteins which alter parameters of cardiomyocyte contraction (1). HCM has a high incidence of more than 1:500 (2, 3), and characteristic features of HCM affecting the heart muscle structure and function have already been extensively investigated (4–7). Typical hallmarks of HCM are variable severity and clinical heterogeneity. They are often associated with imbalanced expression of the mutant and the wild-type allele and a resulting deviation from a 50:50 ratio of mutant to wild-type protein (7–10). Thus, for directed investigation of the pathomechanism of HCM, for design of experimental studies, and, not least, for diagnostic purposes the ability to determine the expression level of mutant protein in the affected tissue is essential. The fraction of mutant protein can be set into relation with results from other examination procedures like mRNA and functional analyses (10, 11) as well as to the severity of the patient's disease at different stages of development (7). Ultimately, it could support personalized treatment development as one diagnostic parameter.

Absolute protein quantification using mass spectrometry (MS) with synthetic stable-isotope-labeled internal standard peptides was developed by Gerber et al. (12) and is widely used (9, 13, 14). To examine expression levels of mutated myosin heavy chain, in our previous studies, (14, 15) we used fairly large tissue samples from human soleus muscle. However, myocardial tissue from genotyped patients with HCM is scarce. Particularly for some HCM-mutations very little human heart tissue is available. The size of heart tissue that can be obtained from endomyocardial biopsies is 1 to 2 mm^3^ according to AHA/ACCF/ESC scientific statement (16). Still, it is of great interest to extract sufficient amount of protein and to reliably determine absolute protein levels even from very small heart tissue samples. Therefore, we asked, whether relative mutant protein levels can be determined with sufficient precision (low noise) and accuracy (low bias), even for single analysis runs with single small, endomyocardial biopsy-sized tissue samples.

To address this question, in this study we took myocardial samples from an explanted HCM patient's heart with point mutation p.Gly716Arg in the ventricular β-myosin heavy chain protein (β-MyHC) and from a control heart. p.Gly716Arg is recorded in *ClinVar database* as pathogenic (17). For samples from four different anatomical regions of the patient's heart we conducted repeated quantifications of the fraction of mutant protein using absolute quantification (AQUA) mass spectrometry with two different strategies. For the first strategy we used large myocardial tissue samples of about 15 mg. For the second strategy we used very small amounts of myocardial tissue of maximum 1 mg with a newly adapted myosin extraction method. By comparing the quantification results for the anatomical regions and the experimental noise for the first strategy to the second strategy we were able to assess the precision of the measurement. Also, by quantifying samples from control myocardium spiked with defined proportions of synthetic peptides we were able to assess the accuracy of the measurement, i.e., how accurate the quantification results reflected the true fractions of mutant protein.

## Materials and Methods

### Tissue Samples

Myocardial tissue samples were collected from an explanted heart of a male HCM patient (32 y). The samples were anonymized. Approval of the local ethics committees and written informed consent for use of the tissue were obtained. The HCM-related mutation p.Gly716Arg was identified in the *MYH7*-gene coding for β-MyHC by a massive parallel sequencing method using a library that included 24 genes related to HCM. Myocardial tissue was dissected from four different regions of the explanted patient's heart: anterior wall of the left ventricle (LV), lateral wall of the right ventricle (RV), left ventricular apex (apex) and right ventricular side of the interventricular septum (IVS). Immediately after dissection the tissue was immersed and stored in liquid nitrogen, as described previously (18, 19). Control myocardium was collected from a non-transplanted heart of a human donor. Mass spectrometric analysis showed no mutation p.Gly716Arg in the control tissue.

### Myosin Extraction

The frozen myocardial tissue pieces were first split into smaller samples in a mortar that was cooled with liquid nitrogen. For further analysis, samples of two different sizes were used. Large tissue samples of about 15 mg were prepared from LV, RV and apex. Myosin from the large samples was extracted as described in Becker et al. (14). Small, endomyocardial biopsy-sized tissue samples ≤ 1 mg were prepared from LV, RV and IVS, and processed separately in a different procedure described here.

**Figure 1:**
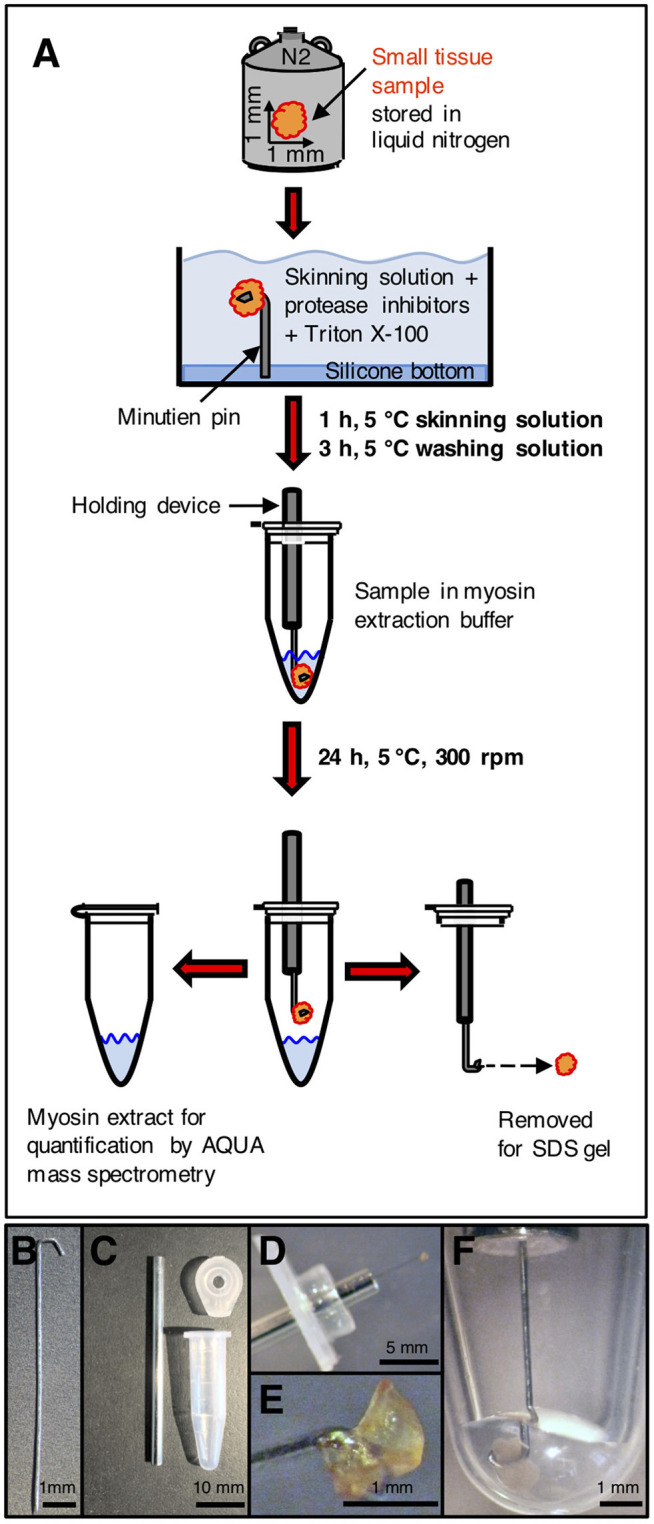
Processing of small tissue sample. **(A)** Small tissue sample was taken from liquid nitrogen storage, immersed in cold skinning solution and attached to a minutien pin. After skinning and washing, the sample on the minutien pin was mounted on a holding device and carefully centered in the extraction buffer in a reaction tube. After extraction, the sample was removed for SDS gel analysis. After precipitation, the extracted myosin was enzymatically digested and used for quantification by AQUA mass spectrometry. **(B)** Minutien pin. Blunt end was formed to a hook. **(C)** Components to place the sample on the minutien pin in the extraction buffer. Left: shortened hypodermic needle filled with silicone gel. Top right: lid of reaction tube with hole drilled in the center of the lid to insert hypodermic needle. Bottom right: 0.6 ml reaction tube without lid. **(D)** Small tissue sample mounted on hooked end of minutien pin. The minutien pin was introduced into the silicone filled hypodermic needle, which was inserted through the lid of the reaction tube. **(E)** Sample mounted on hooked end of the minutien pin. **(F)** Minutien pin with mounted sample in reaction tube with extraction buffer. The sample was completely immersed in the extraction buffer. The minutien pin was fixed in the center to avoid contact of the tissue sample with the tube walls. After myosin extraction the tissue sample could be salvaged without losses and without any remains on the tube walls or in the extraction buffer.

Myosin extraction from small tissue samples is illustrated in Figure 1. Each sample was transferred from liquid nitrogen to an ice-cooled preparation chamber containing skinning solution (18, 19). It was fixed with a minutien pin and the cell membranes were permeabilized (skinned) by addition of Triton X-100 while slightly shaking the chamber. This was to ensure effective penetration of the skinning solution into the tissue. Chemically skinning of the tissue essentially removes soluble proteins without affecting the integrity of the sarcomeres and the cytoskeleton of the cardiomyocytes (20). The whole procedure was performed at 5°C and in the presence of protease inhibitors. These were applied to impede proteolytic degradation of proteins during the skinning procedure and a possible shift in abundance of wild-type and mutant protein. The skinning process was terminated by rinsing with skinning solution without Triton X-100 and without protease inhibitors.

For the extraction of myosin, the minutien pin holding the small tissue sample was fixed on a holder and directly induced into the extraction buffer (modified Hasselbach-Schneider solution (14, 15, 21) containing 500 mM NaCl, 10 mM Hepes, 5 mM MgCl_2_, 2.5 mM ATP, 35 mM DTT, at pH 7.0). Figure 1B through Figure 1F show the holder, the tissue sample fixed on the holder and the tissue sample in extraction buffer. The sample was kept in 5 μl extraction buffer for 24 h at 5°C while spinning at 300 rpm in a shaker (Eppendorf, Thermomixer Comfort) to extract the myosin. The volume of the extraction buffer and the duration of the extraction process were optimized to the size of the tissue sample of about 1 mm^3^. Important was that the extracted myosin had only a small contact area to the inner walls of the reaction tube where adsorption could occur. For all steps we used specific low adsorption tubes (Snap Cap Low Retention Microcentrifuge Tubes, Thermo Scientific, USA) and pipette tips (Low Binding SafeSeal Tips, Biozym, Hessisch Oldendorf, Germany). Time was optimized so that a maximum amount of myosin was selectively extracted from the sample. On termination of the myosin extraction the tissue sample was taken out of the extraction buffer together with the holder and then stored at −80°C or immediately used for analysis in SDS gel electrophoresis. SDS gels contained 12.5 vol% polyacrylamide and proteins were Coomassie stained. Myosin in the extract was precipitated as described in Becker et al. (14). After centrifugation at 14,000 rpm for 15 min, the supernatant was discarded. The myosin sediment was resuspended in 10 μl of 0.1 vol% trifluoroacetic acid (TFA) (J.T.Baker, Avantor, USA), lyophilized by a speed dry vacuum concentrator (Christ RVC 2–18, Osterrode, Germany) and finally solved in 5 μl of 20 vol% acetonitrile (ACN) (Merck KGaA, Darmstadt, Germany) and 0.1 vol% TFA.

### Protein Digestion

Protein concentration was measured by fluorescence quantification with a DeNovix QFX fluorometer (DeNovix Inc., Wilmington, USA). The ratio of protein vs. endoproteinase Lys-C sequencing grade (Roche Diagnostics GmbH, Mannheim, Germany) in digestion buffer (0.1 M ammonium bicarbonate in 10 vol% ACN and 90 vol% H_2_O) (22, 23) was adjusted to 20:1. pH was adjusted to 8.0. Digestion time was 2 × 24 h, whereby fresh enzyme was added after the first 24 h to ensure complete digestion. Desalting of the digest solution was performed with Pierce C18 stage tips (Thermo Fisher Scentific Inc., USA) (24, 25). After desalting, peptides were lyophilized and resuspended in 5 μl of 20 vol% ACN + 0.1 vol% TFA for subsequent mass spectrometric quantification. Peptide adsorption to surfaces was minimized by the addition of 20 vol% ACN as in Becker et al. (14).

### Absolute and Relative Quantification

AQUA mutant and wild-type peptides with identical amino acid sequence to the analogous endogenous peptides were synthesized with stable-isotope-labeling according to Table 1 (PANATecs, Heilbronn, Germany) with a purity of more than 98%. AQUA peptides were added to each extract in exact quantities before digestion (12, 14, 25). 600 fmol of AQUA peptides in the extract volume produced comparable peak areas to the ones of the endogenous peptides in the mass spectrometry chromatograms. Relative quantification of the fraction of mutant peptide vs. wild-type peptide in the sample was performed by AQUA mass spectrometry, as specified in the next section.

**Table 1:** Amino acid sequences of endogenous peptides and of equivalent isotopically labeled AQUA wild-type and mutant peptides used for quantification.

**Peptide**	**Amino acid sequence**	**MW**	**m/z**
Wild-type endogenous	GFPNRILY**G**DFRQRYRILNPAAIPEGQFIDSRK	3909.47	782.89
Wild-type AQUA	GFPNRIL^*^Y**G**DFRQRYRIL^*^NPAAIPEGQFIDSRK	3923.47	785.69
Mutant endogenous	GFPNRILY**R**DFRQRYRILNPAAIPEGQFIDSRK	4008.60	802.72
Mutant AQUA	GFPNRIL^*^Y**R**DFRQRYRIL^*^NPAAIPEGQFIDSRK	4022.60	805.52

*Mass of the peptides (molecular weight, MW) and mass-to-charge ratios (m/z) of the 5 fold ([M+5H^+^]) charged ions in mass spectrometry are listed. Mass-to-charge ratios are shown as average*.

* *Leucine at position 714 and 725 in the amino acid sequence of the β-MyHC was labeled with 6 × ^13^C and 1 × ^15^N heavy isotopes*.

To assess the accuracy of the relative quantification, control experiments with β-MyHC extracted from control myocardium and with defined quantities of synthetically produced mutant and wild-type peptides (PANATecs, Heilbronn, Germany) were performed. The synthetic peptides were not isotope-labeled and therefore could not be distinguished from the endogenous peptides produced by Lys-C digestion from the protein extract. Myosin was extracted from control myocardium as described above. The extracted and digested myosin only showed signals for the wild-type peptide in the mass spectrum, indicating that the control myocardium did not contain the HCM mutation. The extract was divided into aliquots of equal volume. In one aliquot, after digestion the absolute amount of wild-type peptide was quantified by AQUA mass spectrometry. Based on this value, defined quantities of mutant and wild-type synthetic peptides were added to the remaining aliquots before the digestion to adjust defined mutant vs. wild-type ratios. The fractions of mutant and wild-type peptide were quantified by AQUA mass spectrometry and compared to the defined input fractions.

### Mass Spectrometry

Endogenous and added peptides in the digest were separated by an Agilent 1100 capillary LC-system (Agilent Technologies, Santa Clara, USA) with Zorbax SB-C18 (0.5 × 150 mm, 5 μm) analytical column at 5 μl/min. Five microliter digest volume per sample were injected. Liquid-gradient elution was performed with a range of 27.5 vol% to 42.5 vol% acetonitrile with 0.1 vol% TFA in 65 min. The LC-system was connected to a Bruker Esquire 3000 plus electrospray mass spectrometer (Bruker Daltonik GmbH, Bremen, Germany), an ion trap instrument operating in selected ion monitoring (SIM) mode. A narrow mass range (m/z 770 to 815) was scanned to increase the signal-to-noise-ratio. Mutant to wild-type endogenous peptide ratio was determined using the ratio of the peak areas in the extracted ion chromatogram at the respective m/z values for +5-fold charged ions, by taking into account the peak areas of equimolar mutant to wild-type AQUA peptides in the same MS analysis as internal standards (12).

### Statistical Analysis

Values are expressed as mean ± standard deviation. Differences between groups were analyzed by one-way ANOVA and Tukey's test. The level of significance was set at *p* ≤ 0.05. Statistical analysis was performed using *R* (26).

## Results

Here we established a new method for quantifying the fraction of mutant β-MyHC from small, endomyocardial biopsy-sized (1 mm^3^) samples from an HCM patient with a missense mutation in *MYH7*. We compared the results with quantifications from larger tissue samples according to a previously published method (14).

### Myosin Extraction

**Figure 2:**
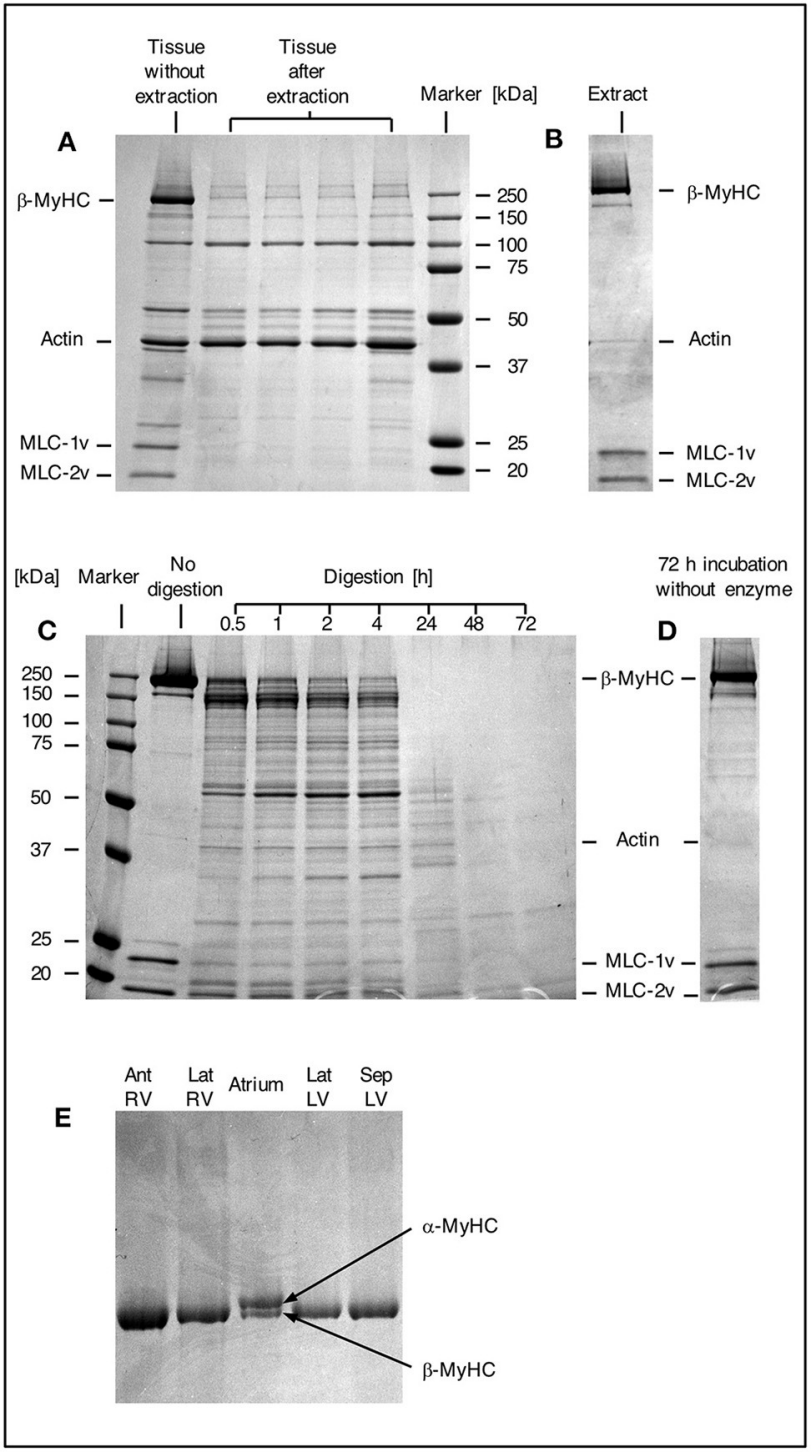
SDS gels. **(A)** SDS gel from small myocardial samples. First lane: myocardial sample which has not undergone extraction process. Lanes 2–5: different myocardial samples after extraction process. Note the prominent β-MyHC and light chain bands (MLC-1v and MLC-2v) in the first lane compared to the other lanes indicating reproducible and high extraction efficiency. **(B)** Proteins in extract after extraction and precipitation. Note the specific extraction of β-MyHC and light chains. Myocardial samples of about 1 mm^3^ each were analyzed. **(C)** First lane: protein marker. Second lane: aliquot from myosin extract immediately after extraction, not exposed to enzyme. Lanes 3–9: aliquots from the same extract, digested in buffer with enzyme for 0.5, 1, 2, 4, 24, 48, and 72 h, respectively. Note that β-MyHC is gradually disappearing with time. Each aliquot had total protein of 3 μg and was digested with 0.1 μg Lys-C. The digestions were started at the same time and stopped at the respective times by addition of 0.1 vol% TFA and freezing in liquid nitrogen. **(D)** Extract aliquot incubated in digestion buffer without enzyme for 72 h. Evidently, β-MyHC and the light chains remain unfragmented without self-degradation. **(E)** SDS gel of different myocardial tissue samples. Lane 1, RV anterior wall, lane 2, RV lateral wall, lane 4, LV lateral wall, and lane 5, LV interventricular septum from the HCM patient with the point mutation p.Gly716Arg. Lane 3 was from *atrium dextrum* (AD) of a donor heart and served as a control for both isoforms α and β-MyHC. In samples from RV and LV only β-MyHC was detectable.

Myosin was extracted from small myocardial samples as described in Figure 1. The efficiency of the extraction process was examined by SDS gel electrophoresis (Figure 2). In Figure 2A a comparison of different small myocardial samples of similar size and consistency before and after myosin extraction is shown. The size of each sample was ~1 mm^3^ (Figure 1E). Lane 1 in Figure 2A demonstrates that the small tissue sample before the extraction process contained ample amounts of β-MyHC with the associated light chains, and actin. After selective extraction of myosin, only very little myosin and myosin light chains were detectable in the small tissue samples (lanes 2 to 5). This demonstrates the high selectivity and efficiency of the myosin extraction. Also, the myosin extraction was highly reproducible for tissue samples of comparable size and consistency. Protein concentrations in the myosin extract were consistently between 0.7 and 1.0 μg/μl (as determined by fluorescence quantification). The extract contained nearly exclusively β-MyHC and associated light chains. Only minute traces of other sarcomeric proteins like actin appeared in the SDS gel (Figure 2B).

### Myosin Digestion

The basis for quantification of mutant β-MyHC protein with amino acid exchange p.Gly716Arg was a specific enzymatic digestion of the protein and mass spectrometric quantification of the peptide of interest containing the mutation. Enzymatic digestion produced a peptide of interest of 33 amino acids, both for wild-type and for mutant protein. Table 1 shows the amino acid sequences and the masses of the mutant and wild-type peptides of interest.

The peptides of interest fulfill the following criteria necessary for mass spectrometric analysis (27, 28): (i) the position of the amino acid exchange is near the center of the peptide and not near the cleavage sites by the enzyme, (ii) both peptides of interest, wild-type and mutant, have a mass suitable for mass spectrometric ionization (Table 1), and (iii) oxidizable amino acids (methionine, tryptophan, and cysteine) are not contained in the amino acid sequence. All the above conditions could be met by using Lys-C as enzyme for digestion (29).

For the determination of the optimum digestion time we used equal aliquots of a myosin extract from cardiac tissue of donor without hypertrophic cardiomyopathy. The aliquots were subjected to different digestion times and analyzed by SDS gel electrophoresis. Figure 2C, lane 2, shows an undigested aliquot. The lanes 3 to 9 show aliquots with different digestion times. The digestions were stopped at the different times and frozen immediately in liquid nitrogen. Figure 2D shows an undigested aliquot incubated in digestion buffer without enzyme for 72 h. It served as a control showing that myosin does not self-degrade even after long incubation times. This confirmed that the protein fragments detected in lanes 3 to 9 were produced by enzymatic cleavage. Already after 0.5 h (lane 3) most of the β-MyHC was digested. After 24 h (lane 7) β-MyHC was not detectable anymore and after 48 h and 72 h (lane 8 and 9) even smaller fragments were further digested. We therefore used a digestion time of 48 h for the quantification.

The α-MyHC isoform, which is usually expressed in atrial myocardium, might also be present in the ventricular myocardium in small fractions of 5–10 % (30). α-MyHC has 93 % amino acid sequence homology to β-MyHC (31) and the analogous peptide of interest differs only in one amino acid. Possible content of α-MyHC in the quantified patient's tissue samples was examined in gel electrophoresis. Figure 2E indicates that α-MyHC was not detectable in the patient's myocardial samples. Also, the α-MyHC specific peptide was not detected in any MS quantification run. Additionally, by simulating Lys-C digestion of the cardiac myofilament protein isoforms and isoforms of cardiac myofilament-associated proteins (32) using the software provided by “ExPASy, the SIB Bioinformatics Resource Portal” (https://web.expasy.org/peptide_mass/, as per November 7, 2019) we confirmed that no other peptides had similar mass or sequence as the peptides of interest.

### Relative Quantification of Mutant Protein by Mass Spectrometry

**Figure 3:**
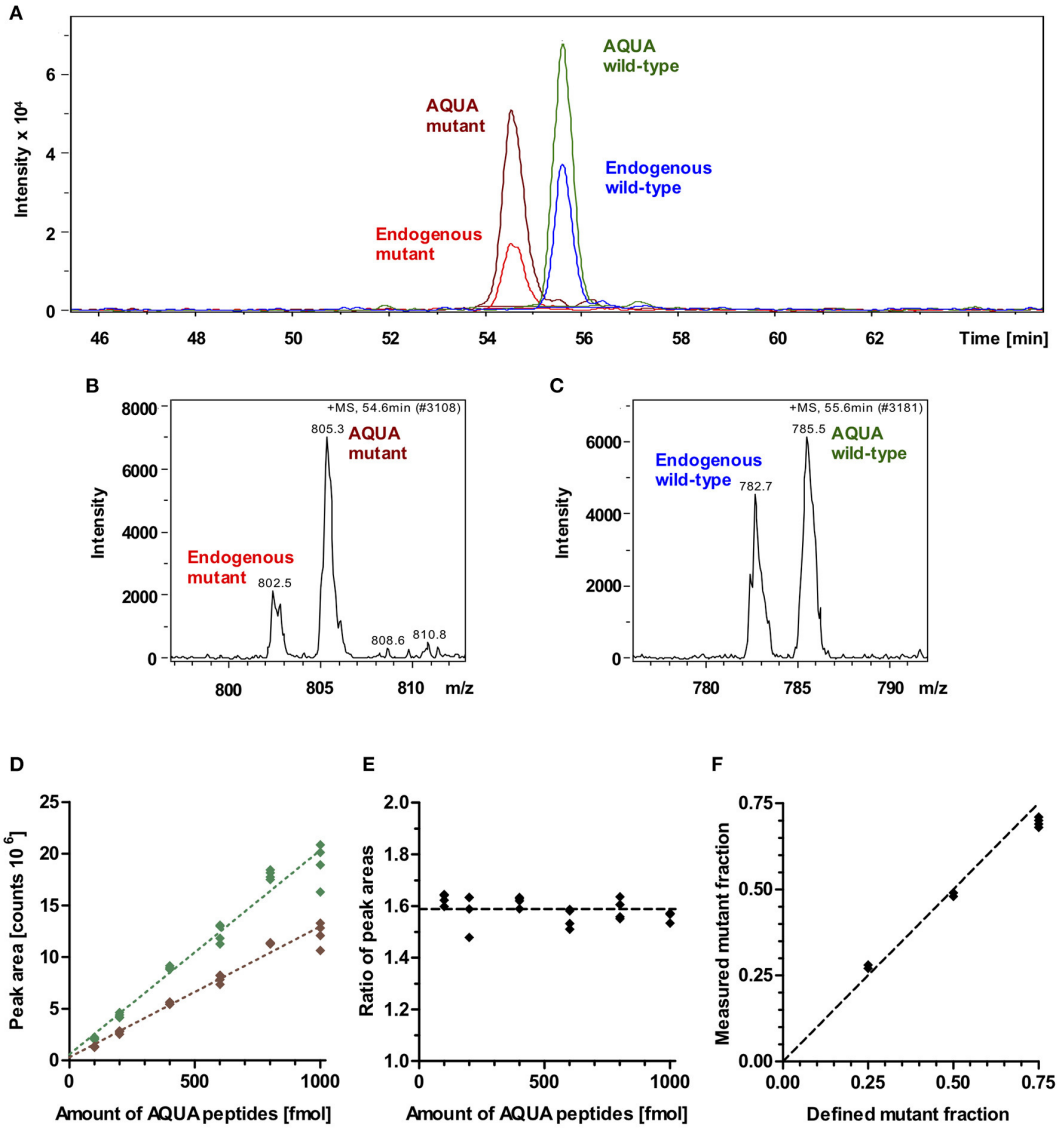
Mass spectrometric quantification of AQUA and endogenous peptides. **(A)** Extracted ion chromatogram of endogenous peptides of interest (wild-type and mutant) and their isotope-labeled analogs (AQUA peptides). AQUA peptides were spiked to the myosin extract in equimolar amounts. Peptides show baseline separation. Therefore, there is no competition in electrospray ionization for the wild-type and mutant endogenous and AQUA peptides. Peak areas were analyzed for quantification. **(B)** Mass spectrum of 5 fold charged ions for endogenous mutant and AQUA mutant peptides. **(C)** Mass spectrum of 5 fold charged ions for endogenous wild-type and AQUA wild-type peptides. **(D)** Absolute measured peak areas in the extracted ion chromatogram for wild-type (green diamonds) and mutant (brown diamonds) AQUA peptides plotted against input amounts of the respective AQUA peptides (100, 200, 400, 600, 800, and 1,000 fmol). Dotted lines represent linear regressions, both with *R*^2^ > 0.99. **(E)** Ratio of measured wild-type to mutant AQUA peptide peak areas at the same input amounts. Dashed line: mean level for all measured values. **(F)** Control experiment. Measured fractions of mutant synthetic peptide which were spiked into myosin extract, plotted over defined input fractions of mutant synthetic peptide. Each data point represents one MS quantification (*n* = 8 quantifications). With perfect accuracy of quantification, the data points would lie on the line of equality (dashed line). The maximum deviation to equality is 0.06.

To quantify the fraction of mutant protein, we employed the established quantitative AQUA mass spectrometry method (12, 14). The peptides used for mass spectrometry are listed in Table 1. The peak areas of the signals for the +5-fold charged ions of the endogenous peptides of interest (mutant and wild-type) and of the analogous synthetic stable-isotope-labeled internal standard peptides (AQUA peptides) were analyzed at the appropriate retention times in the extracted ion chromatogram (Figures 3A–C). AQUA peptides were used as internal standards to ensure correct calibration of the amounts of mutant and wild-type endogenous peptides.

MS-analysis of wild-type and mutant AQUA peptides which were spiked into the myosin extract from control myocardium in fixed equimolar amounts within the range of 100 to 1,000 fmol revealed different peak areas for wild-type and mutant AQUA peptides (Figure 3D). Importantly, peak areas were approximately proportional to the input amounts, and the ratio of wild-type to mutant peak areas remained nearly unchanged over the whole concentration range (Figure 3E). For MS quantification we used AQUA peptide amounts of about 600 fmol. Endogenous peptide amounts were in the same order of magnitude as the AQUA peptide amounts, as shown by the peak intensities in Figure 3A, but varied from sample to sample dependent on the actual size of the sample and on the content of connective tissue in the sample. As long as the endogenous peptide amount is within the range of 100 to 1,000 fmol, we also expect that the quantified ratio of wild-type to mutant endogenous peptides in myosin digests is independent of the extracted amount of myosin. The fraction of mutant endogenous peptide was calculated by normalizing the ratio of endogenous peptide peak areas to the ratio of equimolar AQUA peptide peak areas. It corresponds to the fraction of mutant protein in the tissue sample.

To verify this relative quantification, we performed control experiments with defined input fractions of mutant peptides adjusted by adding synthetic unlabeled wild-type and mutant peptides to myosin extract from control myocardium (see Methods section). Figure 3F shows the measured fractions of mutant peptide compared to the defined input fractions. The maximum difference between measured and defined fractions was 6%, and the root-mean-square deviation was 4.4%, demonstrating a high level of accuracy of the quantification method.

### Quantification Results for Two Different Sizes of Myocardial Tissue Samples

We performed the described relative quantification for (i) large myocardial tissue samples of about 15 mg, and (ii) small, endomyocardial biopsy-sized samples with approximately 1 mm^3^ and maximum 1 mg. Because of fibrosis which is often found in HCM myocardium, the small myocardial tissue samples actually contained more connective tissue than control samples, and the muscle component was effectively smaller than 1 mg. Samples were taken from LV, RV, apex, and IVS (Table 2). Small myocardial tissue samples were analyzed with the new method.

**Table 2:** Quantification results.

**Myocardial region**	**Mean fraction of mutant protein**	**Standard deviation**	**Tissue samples**	***n*** **MS quantifications**
LV large samples	0.329 ^a^	0.031	1	20
LV small samples	0.377 ^d^	0.042	13	16
RV large samples	0.256 ^b^	0.033	1	20
RV small samples	0.324 ^ac^	0.032	11	11
Apex large samples	0.289 ^bc^	0.04	1	13
Septum small samples	0.337 ^a^	0.037	22	40

*Myocardial regions of the HCM patient's heart, respective averages of the fractions of mutant protein from multiple quantifications, standard deviations, number of investigated tissue samples, and numbers of MS quantifications. Large tissue samples allowed more quantifications per sample than small tissue samples. Lowercase superscripted letters behind the mean fractions indicate statistical significance. Mean fractions labeled by a common letter are not significantly different by the Tukey's test at 0.05 level of significance*.

**Figure 4:**
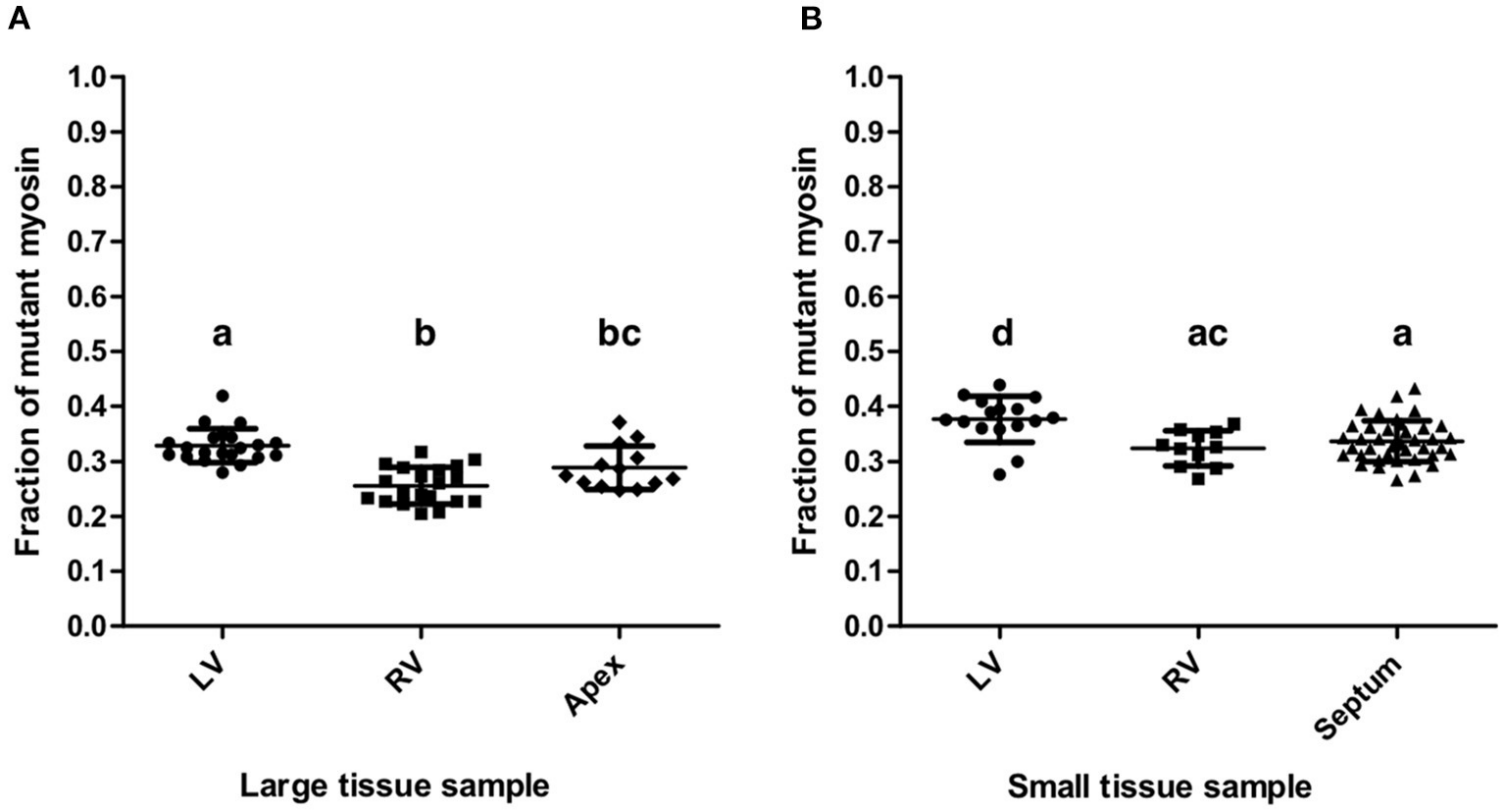
Quantification results. Fractions of mutant myosin by AQUA quantification for large **(A)** and small **(B)** myocardial samples from LV, RV, apex, and interventricular septum. Each data point represents the quantification of one MS-run. Lines indicate means and standard deviations. Lowercase letters above the data groups indicate statistical significance as in Table 2. Means of groups labeled by a common letter are not significantly different by the Tukey's test at 0.05 level of significance.

The mean fraction of mutant β-MyHC for all quantifications from large and small samples of the patient's myocardium was 0.321 ± 0.051, clearly different from a putative 50:50 mutant to wild-type expression level. Quantifications from all large samples had an overall mean mutant β-MyHC fraction of 0.292 ± 0.047 and from all small samples of 0.344 ± 0.041. The difference is statistically significant (*t*-test, *p* < 0.001). Table 2 shows all mean mutant β-MyHC fractions for large and small samples and for all investigated regions. In Figure 4 data points represent the fractions for every individual quantification. The mean fractions differ only by 0.073 for large samples and 0.053 for small samples, with large samples generally having lower fractions than small samples. Interestingly, both in large and in small sample quantifications, the LV samples showed a slightly elevated mutant β-MyHC fraction (Figure 4).

Importantly, the standard deviations of mutant β-MyHC fraction for large samples are only marginally smaller than the ones for small samples (Table 2), further justifying the precision of the new method as in comparison to the old method. The weighted average standard deviation for large samples was 0.034 and for small samples 0.037. Considering the possible systematic bias as evaluated by the control experiment (Figure 3F) and by adding the mean square deviation from the defined fractions to the squared standard deviations of the quantifications, we estimate an error for single measurements of 0.055 in large samples and 0.058 in small samples. Assuming normal distribution of population, these values correspond to the respective 68% confidence interval for a measurement made from a single sample. Accordingly, the 95% confidence interval would be 0.109 and 0.113, respectively. With such precision of quantification it is possible to obtain reliable results about the relative expression level of mutant protein even in single analyses of small tissue pieces from restricted sources or endomyocardial biopsies.

## Discussion

Quantification of the fraction of mutant protein is advantageous for the estimation of disease severity, personalized treatment and prognosis for individual HCM patients. With scarce tissue available we were able to establish a reliable mass spectrometric method for quantification of mutant protein fraction in endomyocardial biopsy-sized samples. By comparing the results of this new method to the conventional method for larger tissue samples (14), accuracy and precision of the quantification could be evaluated.

### Quantification Method for Endomyocardial Biopsy-Sized Tissue Samples

Here we established a protein quantification method for small tissue samples and evaluated the accuracy and the precision by using tissue samples of the explanted heart of an HCM patient with the deleterious mutation p.Gly716Arg in β-MyHC. We employed well established techniques to extract and to digest myosin protein. For quantification of peptides of interest, we used AQUA mass spectrometry in selected ion monitoring (SIM) mode. It has been demonstrated that ion trap MS instruments are suited for AQUA quantification with the SIM method (29).

Attachment of small myocardial tissue samples (endomyocardial biopsy-sized) on minutien pins enabled us to handle each sample in a way that we could remove the whole sample from the extraction buffer in one intact piece. With this separation method no remains of tissue material were left in the extract that could otherwise disturb specific myosin quantification. Extraction was optimized to obtain a maximum yield of extracted myosin from the small samples, which was sufficient for reliable AQUA quantification. Moreover, with the remaining piece of tissue we were able to assert the efficiency of myosin extraction. Tissue samples dissected from the HCM heart were often fibrotic and tissue pieces of dimensions smaller than 1 mm^3^ tended to disintegrate during the mounting procedure. Therefore, we took 1 mm^3^ as the minimum manageable size.

### Accuracy and Precision

We defined the reliability of the quantification method by high accuracy and high precision of the measurements. High accuracy means small systematic errors, and high precision means small random errors. General sources of experimental error stem from myosin extraction, digestion, and AQUA quantification. By comparing small myocardial tissue samples before and after extraction, we saw that the extraction process was very consistent for each sample. At the extraction step no selectivity for either wild-type or mutant myosin is expected, as the single amino acid exchange most likely has no influence on disintegration of myosin filaments.

The total amount of myosin extracted from small myocardial samples was about 5 μg, from large samples about 10 to 20 μg were extracted. The extraction efficiency is comparable to previously published work (33). Each small sample was digested only in one aliquot and underwent the complete sampling, extraction, digestion and quantification process. This means that the quantification error for small samples corresponds to the error of the complete method. On the other side, the extract of each large sample, which was spiked with the AQUA peptides prior to digestion, was split in several aliquots after digestion. Therefore, the quantification error recorded for large samples corresponds only to the experimental error from the processing and measurement steps after aliquotation from the digest. This includes random errors coming from AQUA quantification such as variability of electrospray ionization and sensitivity.

The accuracy of the relative quantification of mutant protein was verified in control experiments, where digests with known fractions of synthetic mutant protein were injected for the AQUA quantification. These control experiments (Figure 3F) showed a maximum deviation of 6% from the true (injected) fractions. This possible systematic error influences the quantification results of both large and small samples.

Random errors are included in the standard deviation of the quantified fractions for each dissected region and for large and small samples (Table 2). All quantification results showed no big difference in standard deviations. This means that neither the region nor the size of the sample had substantial influence on the precision of the quantification.

Nevertheless, the quantifications of the small LV and RV samples resulted in slightly larger mutant protein fractions compared to large LV and RV samples, respectively. The slight differences were statistically significant (Figure 4) but similar to the differences between the four regions of the heart that were analyzed. These differences might reflect a small variability in fraction of mutant β-MyHC throughout the myocardial tissue of the patient.

### Reliability

By repeating measurements, it has been shown that typical quantification errors of AQUA experiments range between 8 and 15 % of the absolute quantified amount (29). Deviations in relative quantification of mutant peptide have to be expected in a similar range. In a study by Helms et al. (9) single quantifications of mutant protein fractions for two different missense mutations in β-MyHC using tissue samples from three different regions of the respective explanted heart showed errors in the range of 2 and 5 %.

Here we demonstrated that accuracy and precision of our quantification method from small tissue samples are as good as for large tissue samples. Quantification results were comparable for every investigated sample within the same error range. Therefore, we conclude that the method is very reliable, even for small, endomyocardial biopsy-sized tissue samples.

Although there is a very small but significant difference between fraction of mutant β-MyHC analyzed with small and large samples, the data show that quantifications of small samples were highly reproducible and reliable, the fraction of mutant β-MyHC varied <5% (Table 2). Hence, the accuracy and the precision of the quantifications allows to conclude that the observed fraction of 32.1% mutant β-MyHC in this HCM patient clearly deviates from a putative balanced expression of 50:50 for mutant vs. wild-type. But here we showed that even a measurement from a single small tissue sample could prove a physiologically significant deviation from a 50:50 protein expression with more than 95% confidence.

Earlier MS quantifications of different missense mutations in *MYH7* (9–11) revealed highly variable mutant β-MyHC fractions ranging from 9% up to 69%. For some mutations it was suggested that the fraction of mutant β-MyHC corresponds to the severity of the disease in these patients. Nevertheless, severity also depends on the primary effects of the mutation on β-MyHC function and other individual modifiers. Interestingly, the HCM patient examined here had significantly <50% mutant β-MyHC in his myocardium. Despite the low mutant fraction the patient was severely affected by HCM, he was transplanted at the age of 32 years. Overall, published data describe a large clinical heterogeneity between individuals (34).

### Diagnostic Implications

In clinical diagnosis of cardiomyopathies and other cardiac diseases, endomyocardial biopsies are taken from different regions of the heart (16). The usual biopsy size (1–2 mm^3^) corresponds to the size of our quantified small tissue samples (~1 mm^3^), where connective tissue was largely excised. Therefore, with only limited quantities of myocardial tissue, like from endomyocardial biopsies, a reliable quantification of the expression of a disease-causing mutation can be performed and put into relation with the severity of the disease. Such quantification from single endomyocardial biopsies could also be of great interest for prognostic statements for patients with familial HCM and for control of the fraction of mutant protein during prospective therapeutic approaches. Also, the here described method is applicable to different mutations in different proteins, as long as the specific protein could be exclusively extracted from the tissue and appropriately digested. Furthermore, this method is also of great interest for quantification of other proteins that are used as markers for heart disease or recovery from disease e.g., in heart failure when the heart is supported with an assist device (35). For this, more sophisticated methods of quantification, as multiple reaction monitoring or parallel reaction monitoring, would even allow protein quantification from digests of whole tissue lysates without the need of specific extraction. Ultimately, we have demonstrated that a specific protein from tiny myocardial biopsy tissue samples can be reliably quantified in a practical way with standard technical procedures.

## Data Availability Statement

The original contributions presented in the study are included in the article, further inquiries can be directed to the corresponding author.

## Ethics Statement

The studies involving human participants were reviewed and approved by Ethics Committee of Hannover Medical School decision no. 2276-2014 from May 27, 2014. The patients/participants provided their written informed consent to participate in this study. Written informed consent was obtained from the individual(s) for the publication of any potentially identifiable images or data included in this article.

## Author Contributions

EB conceived the study, conducted the experiments and wrote the paper. AF supplied patient tissue samples. APi provided mass spectrometry equipment and supervision. APe supplied tissue samples from control subjects. TK supervised the project and revised the manuscript. AR conceived the study, analyzed the data and wrote the manuscript. All authors read and approved the final manuscript.

## Funding

This research was partly supported by a grant from the Deutsche Forschungsgemeinschaft (KR1187/22-1) to TK.

## Conflict of Interest

The authors declare that the research was conducted in the absence of any commercial or financial relationships that could be construed as a potential conflict of interest.

## Publisher's Note

All claims expressed in this article are solely those of the authors and do not necessarily represent those of their affiliated organizations, or those of the publisher, the editors and the reviewers. Any product that may be evaluated in this article, or claim that may be made by its manufacturer, is not guaranteed or endorsed by the publisher.
